# Supplementary material for: Long-Term Management of Post-Stroke Spasticity with Botulinum Toxin: A Retrospective Study
Source: Toxins (Basel). 2024 Sep 3;16(9):383. doi: 10.3390/toxins16090383 (PMC11436082; doi:10.3390/toxins16090383)
Supplement: Supplementary file 1 [file toxins-16-00383-s001.zip › toxins-3164897-supplementary.pdf]

Table S1. Number of patients undergoing treatment with the different brands of BoNT/A in each group.

**Brand**	OnabotulinumtoxinA	AbobotulinumtoxinA	IncobotulinumtoxinA
**SIT**	1	27	20
**LIT**	1	25	21
